# Manifestations of Adipose Tissue Dysfunction

**DOI:** 10.1155/2013/940275

**Published:** 2013-08-20

**Authors:** Nicola Abate, Anne E. Sumner, Manisha S. Chandalia

**Affiliations:** ^1^University of Texas Medical Branch, 301 University Boulevard, Galveston, TX 77555-1060, USA; ^2^Clinical Endocrinology Branch, National Institute of Diabetes, Digestive and Kidney Diseases, National Institutes of Health, Bethesda, MD 20892-1612, USA

Adipose tissue is regarded as an endocrine organ which contributes to regulating systemic energy balance. Since adipocyte is the specialized cell that can safely store triglyceride, normal adipose tissue function in buffering excessive caloric intake is able to prevent systemic metabolic consequences related to high glucose and lipid content in nonadipose cells. Excessive caloric intake relative to energy expenditure is rapidly becoming a feature of most populations around the world, and inability of adipose tissue to fulfill its role is inevitably linked to metabolic abnormalities recognized to increase risk for cardiovascular disease (CVD) and type 2 diabetes (T2D). Several investigators currently advocate focusing on improving our knowledge of mechanisms regulating adipose tissue function independent of adipose tissue mass. Filling this gap in the literature will better enable us to identify novel approaches to treat individuals at risk or with established T2DM and CVD. This special issue is dedicated to various manifestations of adipose tissue dysfunction and, with a combination of clinical papers and reviews of the literature, it addresses various aspects of recent advances and opportunities in this field of research. 

The various papers discuss the existing evidence regarding the role of functional heterogeneity of various adipose tissue areas in determining risk for metabolic complications, such as insulin resistance and dyslipidemia. There is a discussion on recent evidence for adipose tissue inflammation and adipokines production as determinants of systemic metabolic abnormalities of lipid and glucose metabolism. The overall theme is completed by the topics of potential directions for future research and evaluation of the role that maternal nutrition may have in the early development of adipose tissue dysfunction. 

*Nicola Abate* *Nicola Abate* *Anne E. Sumner* *Anne E. Sumner* *Manisha S. Chandalia* *Manisha S. Chandalia*

